# First record of the invasive mosquito species *Aedes* (*Stegomyia*) *albopictus* (Diptera: Culicidae) on the southernmost Mediterranean islands of Italy and Europe

**DOI:** 10.1186/s13071-017-2488-7

**Published:** 2017-11-02

**Authors:** Marco Di Luca, Luciano Toma, Francesco Severini, Daniela Boccolini, Salvatore D’Avola, Diego Todaro, Alessandra Stancanelli, Francesco Antoci, Francesco La Russa, Sandro Casano, Salvatore D. Sotera, Eugenio Carraffa, Veerle Versteirt, Francis Schaffner, Roberto Romi, Alessandra Torina

**Affiliations:** 1 Department of Infectious Diseases, Unit of Vectorborne Diseases, Istituto Superiore di Sanità, Rome, Italy; 2 Department of Veterinary Prevention, Unit of Animal Health Service, ASP Trapani, Pantelleria, Italy; 3 Istituto Zooprofilattico Sperimentale della Sicilia, Laboratory of Entomology and Environmental Vectors Control, Palermo, Italy; 4 City Council of Pantelleria, Pantelleria, Italy; 5 Freelance Veterinarian, Lampedusa, Italy; 6 City Council of Lampedusa and Linosa, Linosa, Italy; 7 Avia-GIS, Risschotlei 33, 2980 Zoersel, Belgium; 8 Francis Schaffner Consultancy, Riehen, Switzerland; 9 Switzerland & Institute of Parasitology, University of Zurich, Zurich, Switzerland

**Keywords:** *Aedes albopictus*, First record, Invasive mosquito, Entry routes, Lampedusa, Linosa, Pantelleria, Italy

## Abstract

**Background:**

*Aedes albopictus*, a known worldwide vector of several mosquito-borne disease pathogens including dengue, chikungunya and Zika viruses, was introduced into Europe in the late 1970s through global trade. First recorded in northern Italy in 1990, this mosquito species has rapidly spread throughout the country, where it was responsible for an outbreak of chikungunya in 2007 that affected more than 200 people. As part of the VectorNet project, which is aimed at improving preparedness and responsiveness for animal and human vector-borne diseases in Europe, a mosquito targeted study was carried out on the three southernmost Italian islands. The objective was to verify the current European southern distribution limits of *Ae. albopictus* and the potential occurrence of other invasive mosquito species, in the light of the introduction of high risk for vector-borne disease pathogens into Europe via migration flows.

**Results:**

In the summer 2015, six surveys for container-breeding mosquitoes were carried out by setting up a network of oviposition traps and BG Sentinel traps in selected areas on the islands of Pantelleria, Lampedusa and Linosa. *Aedes albopictus* was found on all three islands under investigation. The consequences on public health with regard to the presence of this mosquito vector and the migrant people entering the country from Africa and the Middle East are also discussed here.

**Conclusions:**

The detection of the Asian tiger mosquito on these islands, which represent the last European strip of land facing Africa, has important implications for public health policy and should prompt the national authorities to implement tailored surveillance activities and reinforce plans for preparedness strategies in such contexts.

## Background

*Aedes* (*Stegomyia*) *albopictus* (Skuse, 1894) (Diptera; Culicidae), also known as the Asian tiger mosquito, is recognised as one of the 100 most invasive species in the world [1]. The rapid spread of this species is mainly due to passive dispersion by the global trade of used tyres and ornamental plants (such as *Dracaena* spp. or “lucky bamboo”) or by intracontinental transportation in land vehicles [2–8].

In Europe, after its first detection in Albania in 1979 [9], *Ae. albopictus* was reported in Genoa, northern Italy, in 1990 [10] and the Veneto Region in the following year [11].The species has rapidly spread throughout the country with scattered foci mainly in inhabited areas where it now represents the prevalent source of mosquito nuisance [12].

Competent in transmitting a large number of arboviruses worldwide [6, 13], *Ae. albopictus* proved to be responsible for the outbreak of chikungunya (CHIK) fever that occurred in Italy in 2007, which was the first in Europe [14]. The species was also reported as a primary vector in isolated cases of dengue (DEN) and CHIK that occurred in France and Croatia on several occasions [15–19]. Recently, *Ae. albopictus* is also suspected of being involved in Zika (ZIK) virus transmission and its potential as a ZIK virus vector was evaluated in experimental studies with mosquito populations originating from the Americas and Europe [20–22]. In particular, an Italian *Ae. albopictus* population exhibited a partial competence to this virus [21].

These events have raised deep concern in both health authorities and public opinion about the possibility of autochthonous transmission of arboviruses, especially in those European countries where *Ae. albopictus* populations are well-established. Given the invasive behaviour of the mosquito species, and its potential role as a vector of pathogens causing human diseases, surveillance and control strategies are pivotal and should be implemented at a national and local level [23].

Therefore, the widespread presence of *Ae. albopictus* at relevant densities and the high risk of *Aedes*-borne pathogen transmission in Italy have led to the investigation of its occurrence in extreme environments, such as the small Mediterranean islands south of Sicily that represent the last European strips of land facing Africa. Notably, in recent years the introduction of *Ae. albopictus* has been documented in other southwestern Mediterranean islands, such as the Balearic Islands [24] and the Maltese Archipelago [25].

In 2014, with the aim of reinforcing the prevention and control of both animal and human vector-borne diseases, and in improving preparedness and responsiveness for these diseases in the European Union (EU), a joint project with the European Food Safety Agency (EFSA) and the European Centre for Disease Prevention and Control (ECDC), called VectorNet, was set up. One of the main objectives of VectorNet is to implement targeted entomological surveillance/monitoring in Europe and the area surrounding the Mediterranean Basin. In this regard, the Istituto Superiore di Sanità (ISS) of Rome and the Istituto Zooprofilattico Sperimentale della Sicilia (IZS) of Palermo were involved and both institutes carried out an entomological study on the islands of Lampedusa, Linosa and Pantelleria, during the summer of 2015. The study aimed to verify the current southernmost European distribution limits of *Ae. albopictus* and to investigate the potential occurrence of other invasive mosquito species, in light of the presence on Lampedusa of the first reception centre for migrants arriving there. According to the Italian Ministry of Interior, about 11,500 migrants, originating mainly from Africa, arrived in Italy in 2016.

Here we report the first detection of *Ae. albopictus* on these islands.

## Methods

### Study area

**Fig. 1:**
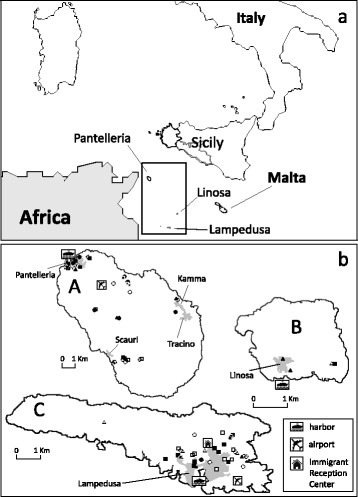
Map of the study area. **a** Geographical locations on the islands of Pantelleria (A), Linosa (B) and Lampedusa (C) in the Mediterranean Sea. **b** Detail of the three islands showing locations of ovitraps (circle), adult traps and human landing catches (square), and larval breeding sites inspected (triangle) during the entomological field study, July-October 2015. Black dots show the findings positive for *Ae. albopictus*, white dots, the negatives

The present study was carried out on the three southern minor islands of Sicily, Lampedusa, Linosa and Pantelleria, in 2015 (Fig. 1a). Lampedusa and Linosa (constituting the Pelagie Archipelago along with the uninhabited islet of Lampione), and Pantelleria represent the southernmost Italian and European areas in front of northern Africa. Lampedusa and Lampione belong geologically to the African continent, whereas Linosa and Pantelleria are of volcanic origin.

Lampedusa and Linosa, with a total area of about 26 km^2^, belong to the Province of Agrigento. Two hundred and five km off the coast of Sicily, Lampedusa is the largest island of the Pelagie Archipelago and the southernmost area of Italy. It has a coastline of 40 km and an area of 20.2 km^2^, extending in length for about 11 km in a west-east direction. The island has 6000 inhabitants, which increases to about 45,000 with the arrival of tourists during the summer season. Lacking rivers and having scarce and intermittent springs, water is supplied almost exclusively from tanks scattered throughout the island by a desalinator facility. Cultivated land generally occupies areas less exposed and more protected from the strong winds, and fields surrounded by dry stone walls or thick hedges of prickly pears are typical on the island. Only three domestic livestock farms are present on the island, two sheep and goat farms and one horse riding stables.

Forty-two km from Lampedusa and 114 km South-East of Pantelleria, Linosa is 3.4 km long and 2.7 km wide, with an area of about 3 km^2^. This small island (which has about 450 inhabitants) is of recent origin (about 300,000 years old) and represents the emerged part of a volcano which has its base at about 800 m below sea level. The highest elevation is Monte Vulcano, which is 195 m a.s.l. Here, the water is available only from tanks.

Pantelleria (which has 7700 inhabitants, increasing to more than 20,000 in summer) belongs to the Province of Trapani and extends for about 80 km^2^. The island is of volcanic origin and lies 110 km southwest of Sicily and 70 km northeast of Tunisia. It has a mountainous terrain, with the highest peak of Montagna Grande (836 m a.s.l.) originating from a volcanic caldera, now covered by woods. There are numerous thermal springs containing silica and sodium carbonate. On Pantelleria there is also a lake of brackish water of volcanic origin, called Specchio di Venere Lake, which contains both meteoric water and thermal spring water (40–50 °C), rich in salt and sulphur compounds. The island lacks sources of drinking water and inhabitants, mainly concentrated in the three villages of Pantelleria, Khamma and Scauri, use water tanks supplied by a desalination plant. The climate is Mediterranean, tempered by sea winds that blow throughout the seasons. The Mediterranean maquis dominates the southeastern part of the island while pine forests grow on the highest peaks, which at lower altitudes are replaced by oak trees; cultivation of vines and olive trees is very common. The horse riding stables is located near the lake; several domestic animal farms (pigs, donkeys, cattle, sheep, goats, poultry), both intensive and household, are scattered on the island, while the only kennel on the island is situated in the village of Pantelleria.

The climate of this Mediterranean area is characterized by a long dry season and a short mild wet season.

### Mosquito collections

**Fig. 2:**
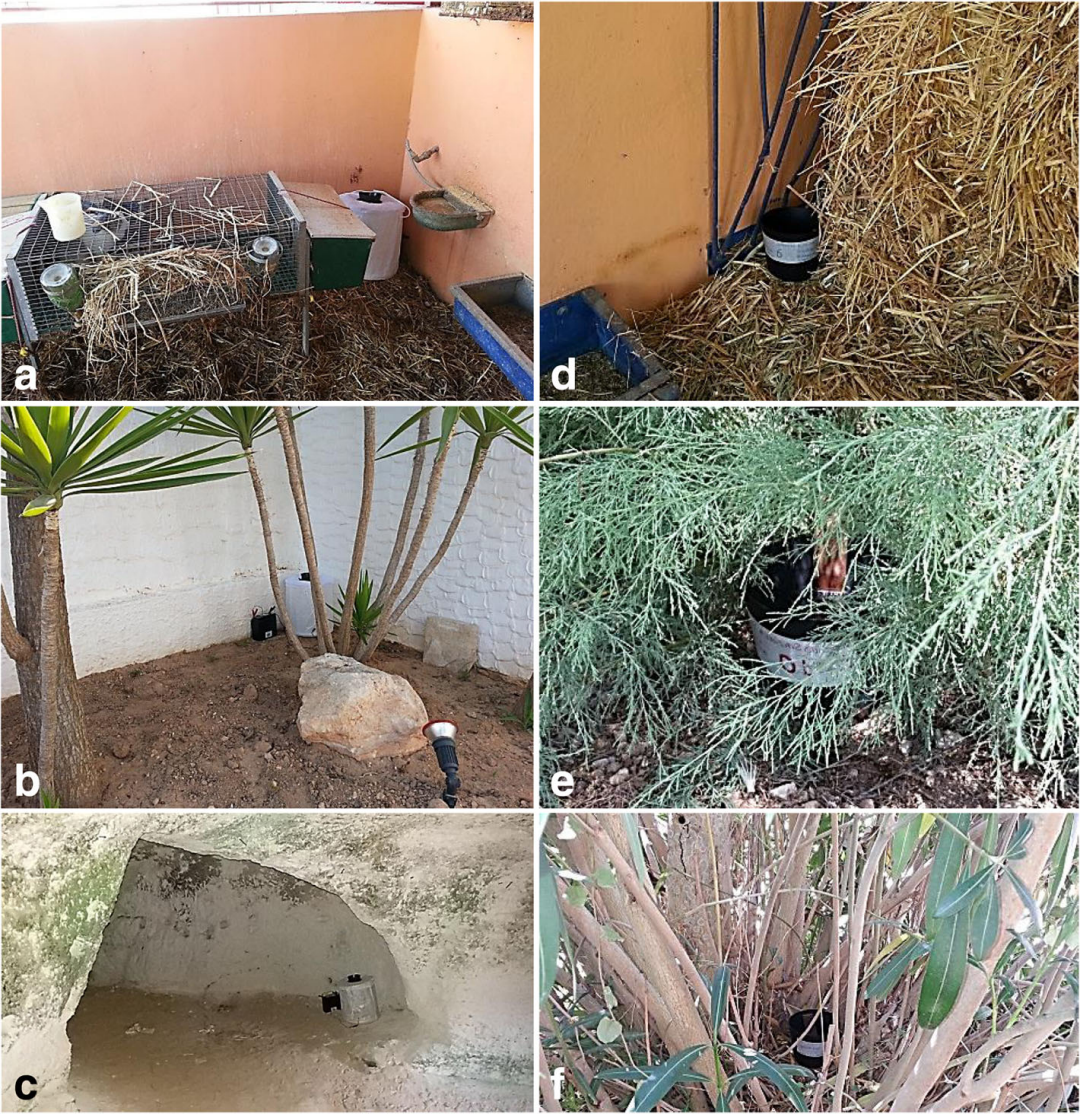
Examples of traps operating at several mosquito collection sites. Odour-baited adult traps (BG-Sentinel) at: **a** Costa House Resort (rabbit hutch); **b** Costa House Resort (dwelling houses); **c** Sanctuary of Madonna di Porto Salvo (cave). Ovitraps in: **d** Costa House Resort (stable); **e** airport (shrub); **f** Sanctuary of Madonna di Porto Salvo (garden)

**Fig. 3:**
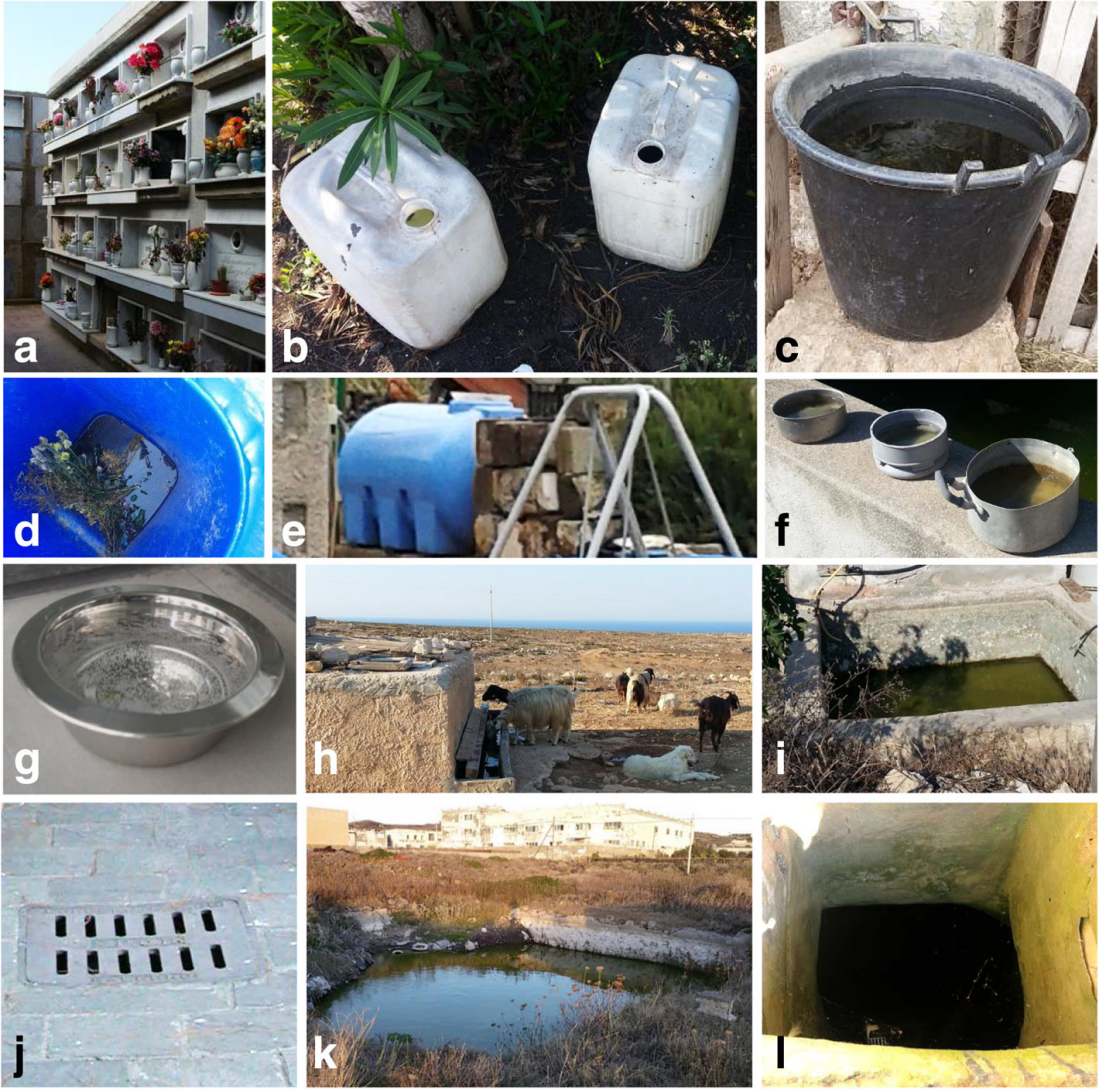
Examples of artificial larval habitats inspected and listed in Table 3: **a** flower vases; **b** plastic jerry cans; **c** bucket; **d** bottom of wheelie dustbin; **e** plastic tank; **f** cooking pots; **g** dog bowl; **h** animal trough; **i** brick-tank; **j** manhole; **k** water pit; **l** well

In the summer and early autumn of 2015, six surveys were carried out on Lampedusa (6–9th and 14–18th July, 27–30th October), Linosa (28th October) and Pantelleria (12–16th and 19–22th October). The monitoring activities were conducted according to ECDC guidelines [26], by setting up a network of BG Sentinel™ traps (Biogents, Regensburg, Germany) and ovitraps in areas frequented by humans and domestic animals (Fig. 2) and by inspecting a series of artificial containers (removable or non-removable), as potential larval breeding sites (Fig. 3), on the islands. Mosquito collection sites are reported in Fig. 1b. Thirty-three ovitraps were placed on Lampedusa and Pantelleria and checked at the end of each survey (3–4 days) for the presence of mosquito eggs and larvae; all collected strips of Masonite from ovitraps were labelled and left to dry at room temperature. Larval collections were performed using dippers or droppers and larvae gathered were preserved in alcohol. Adult mosquitoes were collected by using BG Sentinel™ traps baited with BG-Lure (Biogents, Regensburg, Germany) on Lampedusa and Pantelleria or were directly aspirated by handheld electric aspirators on vegetation or while landing on exposed skin. The traps worked from sunrise to sunset for 1–3 days and were checked every day. All specimens from traps or from human landing catches were frozen. All samples were then transferred to the laboratories of ISS and IZS for identification. Strips of Masonite were examined under a stereo-microscope, eggs were counted and allowed to hatch for rearing in the insectaries of both institutes. Larval and adult specimens were morphologically identified using the keys of Severini et al. [27], Romi et al. [28] and the interactive CD by Schaffner et al. [29].

## Results

*Aedes albopictus* was found on all three of the islands investigated; no other potentially invasive species were identified during the surveys.

During the three surveys on Lampedusa, monitoring activity was carried out by 15 BG Sentinel traps and 12 ovitraps, placed in selected sensitive urban and peri-urban areas of the island, such as shaded verandas and yards of resorts or private dwellings, tyre centres, open green spaces of the hospital, a clinic, the town hall, the Sanctuary of Madonna di Porto Salvo, an isolated place of worship, the airport, the immigrant reception centre, stables and animal shelters (Tables 1 and 2).

**Table 1:** Results of *Aedes albopictus* adult trapping with BG sentinel traps (BG) or handheld electric aspirators (A) on the islands of Lampedusa, Pantelleria and Linosa (July and October 2015)

Island	Latitude (N)	Longitude (E)	Method of capture	Sampling area	Sampling period	No. of adults
Lampedusa	35.50638	12.59555	BG	Cupola Bianca Resort (garden)	06/07/2015	0
	35.50417	12.59528		Sanctuary of Madonna di Porto Salvo	07–09/07/15	1
					27–30/10/2015	24
	35.50500	12.61765		Costa House Resort (rubbit hutches)	07–09/07/15	0
					27–30/10/2015	0
	35.50521	12.61796		Costa House Resort (dwelling houses)	07–09/07/15	0
					27–30/10/2015	0
	35.50420	12.60067		Le Anfore Club Resort	14/07/2015	0
	35.50900	12.61774		Medical Centre	14/07/2015	0
	35.50191	12.60136		Port Restaurant	14/07/2015	9
	35.49887	12.60358		Quarta Isola Restaurant	14/07/2015	0
	35.50437	12.62027		Tyre Centre	15/07/2015	0
	35.50727	12.61354		Private residence (garden)	15/07/2015	7
	35.51299	12.61721		Private residence (garden)	15/07/2015	0
	35.50145	12.61445		Tyre centre	16/07/2015	3
	35.50618	12.60974		Private residence (garden)	16/07/2015	1
	35.50099	12.60067		Private residence (garden)	16/07/2015	0
	35.50478	12.61113		City Hall	17/07/2015	2
	35.51048	12.61708	A	Supermarket forecourt	06/07/2015	2
	35.50338	12.59019		Cala Madonna beach	07/07/2015	1
	35.50417	12.59528		Sanctuary of Madonna di Porto Salvo	07/07/2015	4
					29/10/2015	6
	35.50064	12.60110		Medusa Hotel (garden)	27/10/2015	1
	35.50500	12.61765		Costa House Resort (horse stable)	30/10/2015	1
Pantelleria	36.75917	11.98788	BG	Farm	14/10/2015	0
	36.82745	11.93605		Kennel	15/10/2015	0
					20/10/2015	0
	36.76099	11.99949		Private residence (garden)	19/10/2015	0
	36.81558	11.98921		Private residence (garden)	19/10/2015	4
	36.81931	11.98613		Private residence (garden)	19/10/2015	1
	36.82761	11.94484		Private residence (garden)	20/10/2015	5
	36.83435	11.95353		Private residence (garden)	20/10/2015	4
	36.83112	11.94082		La Nicchia Restaurant	20/10/2015	2
	36.81593	11.98892	A	Lake Specchio di Venere	13/10/2015	1
	36.76088	11.98635		Farm	14/10/2015	1
	36.76108	11.98576		Farm	14/10/2015	1
Linosa	35.86167	12.86577	A	Private residence (garden)	28/10/2015	1

**Table 2:** Placement of ovitraps and numbers of eggs collected on the islands of Lampedusa and Pantelleria, with the respective identification number (ID), georeference, sampling area, entomological survey and number of *Aedes albopictus* eggs found (L: ovitrap lost)

Island	ID	Latitude (N)	Longitude (E)	Sampling area	Sampling period^a^	No. of eggs
Lampedusa	01 L	35.50639	12.59556	Cupola Bianca Resort (garden)	I + II	683
	02 L	35.51048	12.61708	Supermarket forecourt	I + II	20
					V	0
	03 L	35.51046	12.61695	Fruit shop (outside)	I + II	3
	04 L	35.50293	12.60349	L’Aragosta Restaurant (veranda)	I + II	37
					V	0
	05 L	35.50417	12.59528	Sanctuary of Madonna di Porto Salvo	I + II	331
					V	0
	06 L	35.50500	12.61806	Costa House Resort (garden)	I + II	65
	07 L	35.50333	12.62083	New Cemetery	I + II	85
					V	0
	08 L	35.50195	12.60667	Medical Centre	I + II	400
	09 L	35.51167	12.60194	Immigrant Reception Center	I + II	67
	10 L	35.50083	12.61984	Airport	I + II	79
	11 L	35.50333	12.62083	Old Cemetery	I + II	5
	12 L	35.50064	12.60110	Hotel Medusa (garden)	V	12
Pantelleria	01P	36.83011	11.93442	Cemetery of Pantelleria	III + IV	22
	02P	36.83011	11.93442	Cemetery of Pantelleria	III + IV	0
	03P	36.80377	12.03238	Cemetery of Khamma	III + IV	L
	04P	36.75905	11.97651	Cemetery of Scauri	III + IV	0
	05P	36.83469	11.94465	Hospital (car park)	III + IV	39
	06P	36.76088	11.98635	Farm	III + IV	0
	07P	36.80854	11.99058	Farm	III + IV	L
	08P	36.78975	11.97964	Farm	III + IV	31
	09P	36.79397	12.03099	Farm	III + IV	51
	10P	36.75917	11.98788	Farm	III + IV	0
	11P	36.82595	11.94125	Veterinary office	III + IV	0
	12P	36.81445	11.97672	Farm	III + IV	0
	13P	36.82745	11.93605	Kennel	III + IV	0
	14P	36.83066	11.93982	Castiglione Restaurant (veranda)	III + IV	9
	15P	36.76099	11.99949	Private residence (garden)	III + IV	0
	16P	36.79576	11.95997	Basile’s winery	III + IV	31
	17P	36.81558	11.98921	Private residence (garden)	III + IV	19
	18P	36.81931	11.98613	Private residence (garden)	III + IV	12
	19P	36.82761	11.94484	Private residence (garden)	III + IV	0
	20P	36.83435	11.95353	Private residence (garden)	III + IV	0
	21P	36.83112	11.94082	La Nicchia Mare Restaurant (veranda)	III + IV	0

^a^Entomological survey period: I: 6–9 July 2015; II: 14–18 July 2015; III: 12–16 October 2015; IV: 19–22 October 2015; V: 27–30 October 2015

In total 62 adults of *Ae. albopictus* were collected (Table 1). During the inspections of artificial water receptacles (Fig. 3), six larvae were found in flower pots at the cemetery and the sanctuary (Table 3). Several abandoned boats were also inspected in order to find bilge water suitable for the development of native or invasive mosquitoes. At the time of the inspection, these potential breeding sites were found to be dried out.

**Table 3:** Potential man-made receptacles containing water during entomological visits on the islands of Pantelleria, Linosa and Lampedusa

Type of larval breeding site	Lampedusa	Linosa	Pantelleria	Total
	Pos/Neg	Pos/Neg	Pos/Neg	Pos/Neg
Flower vase	4/25	1/15	2/50	7/90
Plastic jerry can	0/0	0/3	0/0	0/3
Bucket	0/3	1/2	1/0	2/5
Wheelie dustbin	0/0	0/0	0/1	0/1
Plastic tank	0/0	0/0	1/1	1/1
Cooking pot	0/0	0/3	0/0	0/3
Dog bowl	0/2	0/0	1/1	1/3
Animal trough	0/3	0/0	0/0	0/3
Brick-tank for rainwater storage	0/3	0/2	0/2	0/7
Manhole	0/0	0/0	1/1	1/1
Water pit	0/0	0/0	0/1	0/1
Well	0/1	0/0	0/0	0/1
Total	4/37	2/25	6/57	12/119

*Abbreviations*: Pos/Neg, no. of positive/no. of negative larval breeding sites for immature stages of *Ae. albopictus*

During the short survey (6 h) on Linosa, two larvae were found in flower pots in the small cemetery and two larvae and one pupa were found in a plastic container below a house gutter in the village (Table 3). In addition, one adult female was collected with an aspirator in the large vegetable garden of a detached house, outside the village (Table 1). No ovitraps or adult traps were placed on the island (Fig. 1b).

Also on Pantelleria a network of traps (*n* = 8) and ovitraps (*n* = 21) was placed at selected sites, such as human dwellings and offices, cemeteries, the hospital, resorts, restaurants, animal shelters, and a kennel (Tables 1 and 2). *Aedes albopictus* was found (19 adults and over 70 immature mosquitoes) both in urban and rural environments. Of eight breeding sites inspected, five were positive for the species: at two of the three cemeteries on the island, two isolated houses with gardens and domestic animals, and a manhole in the yard of the veterinary office (Table 3). Of note, one adult was caught while landing on human bait near the lake, in a typically natural setting (Table 1). During the two surveys, 8 out of 21 ovitraps were found to be positive (Table 2 and Fig. 1b).

## Discussion

Entomological surveys were carried out on three minor Mediterranean islands south of Sicily, Lampedusa, Pantelleria and Linosa, during the summer of 2015. The aim of these surveys was to verify the presence and, in that case, assess the distribution of potentially invasive mosquito species, including *Ae. albopictus*, considered as a competent vector of DEN, CHIK and ZIK viruses. At the beginning of 2015, the VectorNet consortium determined this task as a priority for Italy, not only because of data gaps in the known distribution of *Ae. albopictus*, but also because these areas were identified as high risk for vector-borne pathogen introduction via migration flows. For many years, the southern coasts of Italy have represented the first entrance point for economic migrants and refugees into Europe. The immigrant reception centre of Lampedusa, operating since 1998, represents the primary European entry point for immigrants, mainly from Eritrea, Nigeria, Somalia, Syria, Gambia and Sudan. Although a real risk of transmission of vector-borne pathogens exists on Lampedusa, the extreme climatic and environmental conditions and the short period of stay of the migrants (2–15 days) do not favour the triggering of such events. Moreover, recent studies seem to confirm a low vulnerability at this point of entry. From January 2011 to June 2014, about 49,000 migrants arrived on Lampedusa, but hospitalisation was required for only 378 (0.51%). Among ascertained infectious diseases, mainly respiratory and intestinal distress were reported, while only one case of malaria was detected [30, 31]. Unfortunately, it was impossible to visit the reception centre of Lampedusa, because it operates under military jurisdiction, but the placement of one ovitrap at the entrance of the area was authorized. The site was positive for *Ae. albopictus* in both the surveys in July (60 eggs in the first week and 7 eggs in the second). Similarly, the airport area was monitored, using one ovitrap which was found positive at both trapping occasions in July (70 and 9 eggs, respectively).

This study represents the first entomological investigation focusing on mosquito fauna in the Pelagie archipelago and on Pantelleria. The recording of *Ae. albopictus* on these islands is an important finding, demarcating new boundaries of the distribution range of the species in Europe and indicating Lampedusa as its southernmost limit. As in other similar settings [32–34], it is very difficult to speculate when the Tiger mosquito arrived on these three islands; the mosquito had never been reported there before, probably because of the low density of *Ae. albopictus* populations did not create nuisance for the inhabitants, who did not notice its presence. The maritime route is the most likely pathway, given the high percentage of containers and goods transported by ship and the high number of direct ferries carrying vehicles with tourists from the island of Sicily, where the presence and abundance of *Ae. albopictus* is well-documented [35–37]. This mosquito species could have reached Linosa directly from Sicily or from Lampedusa, due to the daily connections with these two islands.

Despite the fact that the extreme environmental conditions (in terms of aridity, strong winds, seasonal lack of water resources, and scarcity of green areas and breeding sites) do not sustain abundant *Ae. albopictus* populations, they did not, however, prevent the arrival and spread of this invasive mosquito, mainly found in proximity to human settlements. On Lampedusa, the western side is flat and almost completely desert and the isolated residential houses have underground tanks for water storage. The eastern part of the island, where the port and the village are situated, is steadily supplied with water for domestic use and watering, and a wide variety of small and medium water containers, suitable as larval breeding sites, were found. In particular, the highest mosquito densities were found in shaded and cool places, such as the Sanctuary of Madonna di Porto Salvo that is close to an area with several caves, and at the cemetery where a few of the flower pots were colonised by larvae of *Ae. albopictus*, along with larvae of *Culex pipiens.* The survey in late October confirmed the occurrence of *Ae. albopictus*, although at a lower density*.* Due to limitations imposed by the ferry schedules and the inability to load large equipment, the ISS team visited Linosa over a few hours and no traps were positioned. Nevertheless, the inspections carried out in the small touristic village and in some isolated cottages allowed us to find *Ae. albopictus* on the island. Orography, climate and vegetation make Pantelleria the most suitable island to host stable *Ae. albopictus* populations in a large part of its territory. The mosquito was found in several places, though limiting factors, like the strong wind and the fact that the surveys took place late in the season, prevented us from detecting high densities.

## Conclusion

For the first time *Ae. albopictus* was recorded on Lampedusa, Linosa and Pantelleria, where no mosquito surveillance or control programmes are currently implemented. In the light of the presence of this competent vector of important mosquito-borne disease pathogens and the emergency due to the migrant people arriving from Africa and the Middle East, preparedness strategies should be planned on these islands by (i) acquiring further entomological data to monitor the *Ae. albopictus* spread and density and by taking the appropriate control measures, in particular at places at risk of the introduction of pathogens like the migrant reception centre, (ii) implementing a surveillance activity in points of entry (ports and airports) to warn of the introduction of other invasive mosquitoes like *Aedes aegypti*, and (iii) identifying priorities for immediate action and long term needs.
